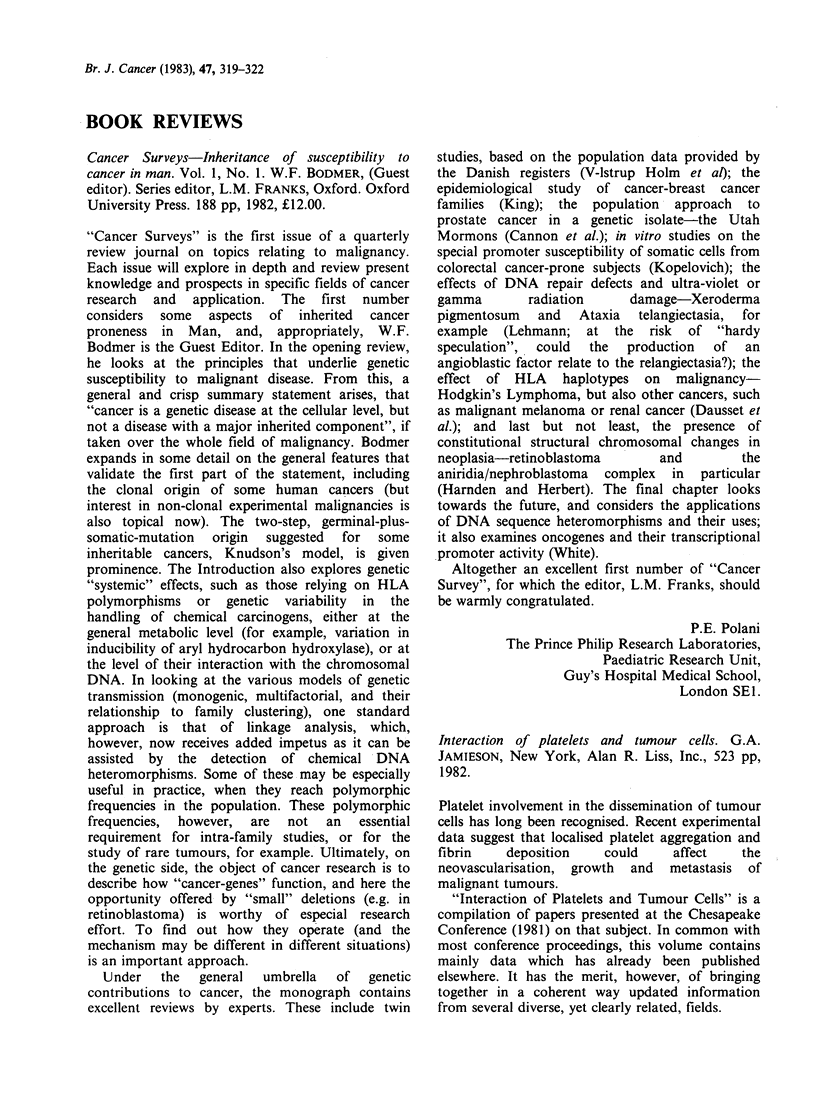# Cancer Surveys—Inheritance of susceptibility to cancer in man

**Published:** 1983-02

**Authors:** P.E. Polani


*Br. J. Cancer* (1983), **47**, 319–322

### BOOK REVIEWS

*Cancer Surveys—Inheritance of susceptibility to cancer in man.* Vol. 1, No. 1. W.F. Bodmer, (Guest editor). Series editor, L.M. Franks, Oxford. Oxford University Press. 188 pp, 1982, £12.00.

"Cancer Surveys" is the first issue of a quarterly review journal on topics relating to malignancy. Each issue will explore in depth and review present knowledge and prospects in specific fields of cancer research and application. The first number considers some aspects of inherited cancer proneness in Man, and, appropriately, W.F. Bodmer is the Guest Editor. In the opening review, he looks at the principles that underlie genetic susceptibility to malignant disease. From this, a general and crisp summary statement arises, that "cancer is a genetic disease at the cellular level, but not a disease with a major inherited component", if taken over the whole field of malignancy. Bodmer expands in some detail on the general features that validate the first part of the statement, including the clonal origin of some human cancers (but interest in non-clonal experimental malignancies is also topical now). The two-step, germinal-plus-somatic-mutation origin suggested for some inheritable cancers, Knudson's model, is given prominence. The Introduction also explores genetic "systemic" effects, such as those relying on HLA polymorphisms or genetic variability in the handling of chemical carcinogens, either at the general metabolic level (for example, variation in inducibility of aryl hydrocarbon hydroxylase), or at the level of their interaction with the chromosomal DNA. In looking at the various models of genetic transmission (monogenic, multifactorial, and their relationship to family clustering), one standard approach is that of linkage analysis, which, however, now receives added impetus as it can be assisted by the detection of chemical DNA heteromorphisms. Some of these may be especially useful in practice, when they reach polymorphic frequencies in the population. These polymorphic frequencies, however, are not an essential requirement for intra-family studies, or for the study of rare tumours, for example. Ultimately, on the genetic side, the object of cancer research is to describe how "cancer-genes" function, and here the opportunity offered by "small" deletions (e.g. in retinoblastoma) is worthy of especial research effort. To find out how they operate (and the mechanism may be different in different situations) is an important approach.

Under the general umbrella of genetic contributions to cancer, the monograph contains excellent reviews by experts. These include twin studies, based on the population data provided by the Danish registers (V-lstrup Holm *et al*); the epidemiological study of cancer-breast cancer families (King); the population approach to prostate cancer in a genetic isolate—the Utah Mormons (Cannon *et al.*); *in vitro* studies on the special promoter susceptibility of somatic cells from colorectal cancer-prone subjects (Kopelovich); the effects of DNA repair defects and ultra-violet or gamma radiation damage—Xeroderma pigmentosum and Ataxia telangiectasia, for example (Lehmann; at the risk of "hardy speculation", could the production of an angioblastic factor relate to the relangiectasia?); the effect of HLA haplotypes on malignancy—Hodgkin's Lymphoma, but also other cancers, such as malignant melanoma or renal cancer (Dausset *et al.*); and last but not least, the presence of constitutional structural chromosomal changes in neoplasia—retinoblastoma and the aniridia/nephroblastoma complex in particular (Harnden and Herbert). The final chapter looks towards the future, and considers the applications of DNA sequence heteromorphisms and their uses; it also examines oncogenes and their transcriptional promoter activity (White).

Altogether an excellent first number of "Cancer Survey", for which the editor, L.M. Franks, should be warmly congratulated.

P.E. Polani
The Prince Philip Research Laboratories,
Paediatric Research Unit,
Guy's Hospital Medical School,
London SE1.